# Tobacco industry globalization and global health governance: towards an interdisciplinary research agenda

**DOI:** 10.1057/palcomms.2016.37

**Published:** 2016-07-05

**Authors:** Kelley Lee, Jappe Eckhardt, Chris Holden

**Affiliations:** 1 Simon Fraser University, Burnaby, Canada; 2 Department of Politics, University of York, York, UK; 3 Department of Social Policy and Social Work, University of York, York, UK

## Abstract

Shifting patterns of tobacco production and consumption, and the resultant disease burden worldwide since the late twentieth century, prompted efforts to strengthen global health governance through adoption of the Framework Convention on Tobacco Control. While the treaty is rightfully considered an important achievement, to address a neglected public health issue through collective action, evidence suggests that tobacco industry globalization continues apace. In this article, we provide a systematic review of the public health literature and reveal definitional and measurement imprecision, ahistorical timeframes, transnational tobacco companies and the state as the primary units and levels of analysis, and a strong emphasis on agency as opposed to structural power. Drawing on the study of globalization in international political economy and business studies, we identify opportunities to expand analysis along each of these dimensions. We conclude that this expanded and interdisciplinary research agenda provides the potential for fuller understanding of the dual and dynamic relationship between the tobacco industry and globalization. Deeper analysis of how the industry has adapted to globalization over time, as well as how the industry has influenced the nature and trajectory of globalization, is essential for building effective global governance responses. This article is published as part of a thematic collection dedicated to global governance.

## Introduction

The marked shift in tobacco-related disease and death, from traditional to emerging markets in low and middle-income countries (LMICs), began to garner major attention within the public health community during the 1980s (Stebbins, 1987). The 1983 World Conference on Smoking and Health, for example, was the first to give major attention to the increased targeting of “Third World countries” by tobacco companies. The expansion of transnational tobacco companies (TTCs) into Latin America from the 1960s, newly industrialising economies in Asia during the 1980s, and Eastern Europe, the Middle East and Africa from the 1990s also raised growing concerns (Nichter and Cartwright, 1991; Connolly, 1992; MacKay, 1992). The creation of the World Health Organization (WHO) Programme on Tobacco or Health in 1990 was underpinned by a reframing of tobacco use, from a focus on individual lifestyle choices, to societal factors that shape risk behaviours (Balbach *et al*., 2006). This latter perspective intensified collective action by WHO member states, initially by “documenting the scale of the tobacco epidemic” (Boucher, 2000), and then negotiating the Framework Convention on Tobacco Control (FCTC) (Collin *et al*., 2002).

The FCTC is rightfully lauded as a key marker in emerging forms of global health governance (GHG). Adopted in May 2003, and entering into force in February 2005, the international treaty boasts 180 states parties (nearly 90% of the world’s population) as of May 2016. During the first decade of implementation, the treaty has led to important achievements. Financial support from the Bloomberg Philanthropies, and Bill and Melinda Gates Foundation, has funded a WHO-led effort to implement six measurable and proven tobacco-demand reduction measures known by the acronym MPOWER. Data on tobacco consumption has also been significantly improved through the four surveys of the Global Tobacco Surveillance System—Global Youth Tobacco Survey, Global School Personnel Survey, Global Health Professions Student Survey, and Global Adult Tobacco Survey—developed by the US Centers for Disease Control, WHO and Canadian Public Health Association. This data has enhanced capacity in LMICs to design, evaluate and report on the implementation of key FCTC articles (Ng *et al*., 2014). Celebrating the FCTC’s tenth anniversary in 2015, WHO Director-General Margaret Chan cited 7.3 million deaths averted because of the adoption of at least one high-impact demand reduction measure in 41 countries (Chan, 2015).

Importantly, fuller understanding of tobacco industry activities supported these efforts, substantially enabled by the public release (through whistleblowers and US litigation) of internal tobacco industry documents beginning in the 1990s (Hurt *et al*., 2009). Alongside a WHO inquiry (WHO, 2000), and reports by investigative journalists (Beelman *et al*., 2000) and civil society organizations (Campaign for Tobacco Free Kids/ASH, 2001), painstaking analyses of millions of documents by public health researchers revealed industry tactics and strategies for expanding worldwide (Glantz *et al*., 1996; Lee *et al*., 2012b; Gilmore *et al*., 2015). This evidence has been fundamental to negotiating and implementing the FCTC.

Despite these achievements, and discussion of a tobacco “endgame” (Novotny, 2015), production and consumption continues to grow worldwide. While global adult (aged 15+ years) smoking prevalence has declined, from 29% in 1995 (Jha *et al*., 2002) to 21.1% in 2013 (WHO, 2015), aggregate data obscure increases in certain populations such as young females and indigenous peoples. Moreover, because of growing populations, the absolute number of users and volume consumed are increasing (Ng *et al*., 2014). In 2014 there were 1 billion smokers worldwide, consuming 5.8 trillion cigarettes, resulting in 6 million tobacco-related deaths annually (Eriksen *et al*., 2015). This is projected to rise to 1.6 billion smokers by 2025 (Bilano *et al*., 2015), causing 8 million deaths annually by 2030 (WHO, 2015). Behind these trends is a buoyant industry that has continued to enjoy growth and profitability. In 2015, the industry experienced its best year by volume sales since 2006, and TTCs have boasted record highs in share prices and financial returns since 2011 (Wachman, 2012; Gara, 2014; Banjo, 2016). The illicit tobacco trade has also thrived during the same period, embedded within a network of criminal activity with global reach (FATF, 2012). As Fooks (2014) writes, “despite growing regulatory risks there has always been a tendency to exaggerate news of the industry’s demise.”

In this context, this article argues that collective action to stem the tobacco pandemic requires fuller understanding of the nature and dynamics of tobacco industry globalization. We begin by reviewing the existing public health literature on tobacco industry globalization, identifying how globalization is defined and measured, historically located, conceptualised by unit and level of analysis, and ascribed with power. On the basis of the findings of this review, we draw on the disciplines of business studies and international political economy (IPE) to set out an interdisciplinary research agenda. We argue that this expanded understanding of tobacco industry globalization is a prerequisite to strengthening GHG.

## Tobacco industry globalization: a review of the public health literature

We searched the peer-reviewed public health literature on tobacco industry globalization, published from 1980–2016, during March–April 2016 using PubMed (which includes citations and abstracts from the fields of biomedicine and health, covering portions of the life sciences, behavioural sciences, chemical sciences, and bioengineering). Searches were conducted using the keywords “globali*ation” AND “tobacco” AND “industry” (*n* = 490); “transnational” AND “tobacco” AND “industry” (*n* = 106); and “globali*ation” AND “transnational” AND “tobacco” (*n* = 57). Articles primarily concerned with tobacco use, or tobacco-related morbidity and mortality, were excluded. Articles related to the tobacco industry’s pursuit of globalization, or how the industry has sought to influence globalization, were included. After duplicates were removed, the remaining 76 papers were coded along four dimensions (see supplementary Table 1): definitions and measures of globalization; analytical timeframe; units and levels of analysis; and nature of power amid tobacco industry globalization.

### Definitions and measures of globalization used

While an explicit definition of globalization is not provided in most of the articles reviewed, each associates tobacco industry globalization with one or more of the following trends.

First, 44 articles (58%) associate globalization with increased *policy influence* of the industry. Most papers describe how TTCs undermined national-level tobacco control policies in Europe (Hilamo, 2003; Szilagyi and Chapman, 2003a, 2004; Gilmore *et al*., 2007; Krasovsky, 2010; Shrinae *et al*., 2012; Lunze and Migliorini, 2013; Skafida *et al*., 2014), Asia (Chantornvong *et al*., 2000; Knight and Chapman, 2004a; MacKenzie *et al*., 2004; Tong and Glantz, 2004; Zhong and Yano, 2007; MacKenzie and Collin, 2008; Muggli *et al*., 2008; Charoenca *et al*., 2012), the Middle East (Nakkash and Lee, 2009), Latin America (Sebrie *et al*., 2005, 2009; Holden and Lee, 2011) and Africa (Curry and Ray, 1984; Stebbins, 1987; Otanez *et al*., 2009; Delobelle *et al*., 2016). A few studies focus on policy influence at the regional and global levels through industry bodies (Ong and Glantz, 2000; McDaniel *et al*., 2008), trade and investment agreements (Holden and Lee, 2011; Fooks and Gilmore, 2013; Crosbie *et al*., 2014; Eckhardt *et al*., 2015), public health bodies (Weishaar *et al*., 2012; Peeters *et al*., 2016; Smith *et al*., 2016b) and religious groups (Petticrew *et al*., 2015).

Second, 30 articles (39%) define globalization as TTC *marketing* activities in emerging markets. Moodie *et al*. (2013) describe transnational corporations as “major drivers of global epidemics of NCDs”, including the “sale and promotion of tobacco” in LMICs. This has been achieved through sophisticated marketing strategies promoting western lifestyles, and developing products for new markets (Szilagyi and Chapman, 2004; Hafez and Ling, 2005; Gilmore, 2012; Delobelle *et al*., 2016). Marshall (1991) attributes the shift in Oceanic island countries since the 1980s, from “home-grown and twist tobacco” to commercially manufactured cigarettes, to aggressive marketing. Ethnographic research in Argentina, Chile, Ecuador and Peru concludes that “TTC’s marketing strategies override cultural differences in the choices people make regarding smoking and health” (Stebbins, 2001). Internal documents reveal the specific strategies to achieve this. Knight and Chapman (2004b) examine how TTCs used the themes of music, entertainment, adventure, sport, glamour and independence “to construct a tobacco culture” among young Asians. TTCs in Japan “developed a lucrative market for mild, light, and ultra-low-tar cigarettes” by playing on the concept of keihaku tansho (light-thin-short-small) (Assunta and Chapman, 2007), and using Hollywood film stars (Lambert *et al*., 2004). Stanton *et al*. (2010) document how BAT “sought to transfer values associated with [London’s Ministry of Sound] lifestyle brand” to China and Taiwan. Where necessary, TTCs adapted marketing strategies to local contexts and targeted specific populations, notably females and youth (Lunze and Migliorini, 2013). In South Korea, British American Tobacco (BAT) undertook market research to “understand consumer preferences, cultural characteristics and social changes affecting women and girls” (Lee *et al*., 2009). TTCs overcame “entrenched cultural and institutional barriers” by identifying youth as “more favourably inclined towards imported brands”, and using new distribution channels and promotional activities. Japan Tobacco International (JTI) (Honjo and Kawachi, 2000) and KT&G (Lee *et al*., 2012a) responded to foreign competition by mimicking their marketing strategies.

Third, 25 papers (33%) associate globalization with *market access and growth* by TTCs in emerging markets. In many cases, this was achieved through the above described policy influence encouraging countries to liberalize tobacco trade and investment, privatize state-owned enterprises, and pursue joint ventures. This literature describes TTCs expanding through takeovers in Latin America from the 1960s (Stebbins, 1994), leaf growing in Africa from the 1970s (Curry and Ray, 1984; Otanez *et al*., 2009), and pressuring Asian markets to open from the 1980s (Connolly, 1992; Lee *et al*., 2013; MacKenzie *et al*., 2015). For example, Gultekin-Karakas (2015) examines how “the liberalisation process facilitated by the state under the auspices of international institutions … paves the way for market expansion” of TTCs in Turkey. The aggressive expansion of TTCs in Eastern Europe is ascribed to liberalization, coinciding with the end of the Cold War (Gilmore and McKee, 2004b; Gilmore *et al*., 2005; Gilmore *et al*., 2007).

Other analyses focus on how privatization (Gilmore *et al*., 2005; Nakkash and Lee, 2008; Hurt *et al*., 2012; Gultekin-Karakas, 2015), tariffs and taxation (Szilagyi and Chapman, 2003a; Gilmore *et al*., 2007; Krasovsky, 2010; Holden and Lee, 2011; Shrinae *et al*., 2012), intellectual property rights and investor-state dispute settlement mechanisms (Fooks and Gilmore, 2013), have been used to facilitate TTC market access and growth (Drope and Chavez, 2015). The illicit trade is also described as part of TTC strategies, with complicity in large-scale cigarette smuggling extending into Eastern Europe (Gilmore and McKee, 2004a; Skafida *et al*., 2014), Asia (Lee *et al*., 2004; Lee and Collin, 2006; Lee *et al*., 2008), Africa (LeGresley *et al*., 2008) and the Middle East (Nakkash and Lee, 2008). Smuggling circumvented import bans and quotas in restricted markets, helped to undermine regulation, and build brand presence ahead of market opening.

Fourth, four articles (5%) associate globalization with *structural consolidation* of the industry, although none provide detailed analysis. Yach and Bettcher (2000: 207) describe how “mega mergers and acquisitions have dramatically changed the face of the worldwide cigarette industry” and created “an increasingly globalised marketplace”. Similarly, Bialous and Peeters (2012) examine a 20-year period from the early 1990s as “marked by mergers and acquisitions [M&As] that led to the existence, today, of four major transnational tobacco companies”. Holden and Lee (2009) write that the industry “operates in an essentially oligopolistic fashion, and the market positions of TTCs are strongly protected by barriers to entry”.

It is because of the absence of clear definition, perhaps, that the existing literature does not measure tobacco industry globalization in any meaningful way. Where globalization is associated with *policy influence*, for instance, the indicator is whether policies favourable or unfavourable to industry interests (for example, tax and tariff rates) are supported by policy makers. For example, Szilagyi and Chapman (2004) argue that, along with “early participation in the privatisation of the former state tobacco monopoly”, there was a “well orchestrated industry effort to influence decision makers to avoid strict regulation” in Hungary. Hurt *et al*. (2012) cite two favourable amendments in less than 4 years, by the Indonesian Government Regulation on the Tobacco Control Act, as evidence of TTC influence. However, these papers are primarily concerned with the political power of TTCs in various policy settings, which may or may not be related to globalization per se. Papers which define globalization as *marketing* cite specific marketing practices, targeted populations, and changes in tobacco consumption. For instance, Chu *et al*. (2011) describe how BAT and Philip Morris International (PMI) studied the role of gift giving in Chinese custom and adapted marketing of their brands accordingly. Papers defining globalization as *market access and growth* largely refer to increased presence and market share in a given country. For example, Lee *et al*. (2009) analyse South Korea following market liberalization, and found TTCs increased market share from 2.9% in 1988 to 41.7% in 2009. Acquisitions of kretek manufacturers by PMI and BAT are used as indicators by Hurt *et al*. (2012) that “TTCs have now successfully penetrated the Indonesian cigarette market” and begun “their Westernized transformation”.

### Analytical timeframe of tobacco industry globalization

We looked at the timeframes in which tobacco industry globalization is analysed in the public health literature. While 88% of the reviewed literature has been published since 2000, 3 papers (4%) locate their analysis from the 1960s; 5 (7%) from the 1970s; 18 (24%) from the 1980s; 43 (57%) from the 1990s and 7 (9%) from the 2000s onwards. The desire for evidence to support the FCTC process, which commenced in the late 1990s, may have focused greater research efforts on this time period. The public release of millions of internal industry documents also explains the temporal focus of the existing literature. The capacity to search digital copies of these documents, via the creation of on-line archives during the 2000s, greatly enabled new scholarship (Hurt *et al*., 2009). However, the collections currently hold few documents dating later than the early to mid 2000s.

### Units and levels of analysis

The *unit of analysis* in social research refers to the major entity (the “who” or “what”) analysed from which data is gathered. *Level of analysis* concerns the location, size or scale of a research target. Together, the unit and level of analyses help define the population of a research enterprise.

Fifty-five (72%) of the papers reviewed (and 80% of papers published since 2000) use TTCs as the unit of analysis.^1^ Detailed descriptions of TTC activities are painstakingly gleaned from internal documents, providing valuable glimpses of the activities of specific companies. This literature offers analysis of how the industry is changing, often in specific groups of countries or regions, and TTC adaptation accordingly. A good example is Gilmore (2012) who documents “how the global tobacco market has changed, how [TTCs] are responding and the implications for tobacco control”. Similarly, Bialous and Peeters (2012) discuss the “large number of privatisations, mergers and acquisitions [M&As] that served to strengthen the position of the four largest TTCs” since the early 1990s. For the most part, this research treats TTCs as part of an increasingly homogeneous industry in pursuit of globalization. For example, Yach and Bettcher (2000: 207) describe “how the tobacco industry operates as a global force, regarding the world as its operating market by planning, developing, and marketing its products on a global scale”. They describe the “homogenisation of the global tobacco industry and the creation of a new global shared culture enshrined in the concept of a global smoker” (Yach and Bettcher, 2000: 207). With the accumulation of detailed knowledge about TTC activities over time, scope for comparative analysis of TTC activities is now possible. As Gilmore (2012: 124) writes, “While the global market context is identical for all TTCs, there may be differences in the nature of each company’s response”.

Seventeen of the reviewed papers (22%) focus on the industry, with much of this scholarship published before 2000. Most of this work sets out broad trends in the industry, in terms of, its structural consolidation and expansion into LMICs. For the most part, this research is descriptive in nature, and offers limited explanation in relation to globalization of the world economy.

Four papers (7%) use the state as the unit of analysis. For example, Chantornvong *et al*. (2000: 913) apply political mapping and stakeholder analysis to understand the political and economic context for tobacco control in Zimbabwe and Thailand. They conclude that the policy environments “are clearly being shaped by developments in the global political economy, which means that efforts to strengthen national control policies need to be set within the context of globalization”. Lunze and Migliorini (2013) conduct a state-focused analysis of TTC influence of tobacco control policy in Russia since the 1990s. Baker *et al*. (2014) analyse how international and regional trade regimes have facilitated increased market penetration by TTCs, and driven increased tobacco consumption in Asian countries. Eckhardt *et al*. (2015) retain a focus on the state unit, but extend analysis to trends or patterns in the positions of World Trade Organization (WTO) members on tobacco control measures.

The levels of analysis of the papers reviewed are distributed as follows: 45 (59%) are national; 6 (8%) are regional and 24 (32%) are global.^2^ The geographical distribution of papers for national and regional level analyses is Asia (24), Europe (13), Latin America (6), Africa (3) and the Middle East (3). Stebbins (1994) unusually brings together “macro-level and micro-level implications of the tobacco companies’ promotions” with smoking behaviours by secondary school students in Mexico and Guatemala.

### Agency versus structural power in tobacco industry globalization

Farnsworth (2004) distinguishes between the agency and structural power of corporations. *Agency power* is the capacity of corporations to act independently in ways that achieve desired ends. Firms may exert agency power through various forms of political engagement and institutional participation. *Structural power* operates in situations where governments are compelled to favour industry interests without the need for firms to take explicit action. Farnsworth and Holden (2006) write that globalization tends to increase corporate structural power by increasing the mobility of capital. Since investment is a fundamental source of production, employment, consumption and, by extension, tax revenues, the opportunity for corporations to move operations out of a national economy may compel the government to act in ways amenable to corporate interests.

The focus, in 72 (95%) of the reviewed papers, is on the exertion of agency power by the tobacco industry to gain favourable outcomes. This is consistent with the prevailing definition of tobacco industry globalization as policy influence and TTCs as the primary unit of analysis. Many papers document the use of agency power in specific national settings to influence science and policy (for example, Hilamo, 2003; Szilagyi and Chapman, 2003b; Gilmore *et al*., 2005; Gilmore *et al*., 2007; Sebrie *et al*., 2009; Charoenca *et al*., 2012; Shrinae *et al*., 2012; Lunze and Migliorini, 2013, Holden *et al*., 2010b). Others analyse agency power exerted at regional (Holden and Lee, 2011; Peeters *et al*., 2016) or international levels (Zhong and Yano, 2007; Holden *et al*., 2010a; Weishaar *et al*., 2012; Crosbie *et al*., 2014; Eckhardt *et al*., 2015), as well as through third parties (Tong and Glantz, 2004; Mamudu *et al*., 2008; McDaniel *et al*., 2008; Muggli *et al*., 2008; Petticrew *et al*., 2015; Smith *et al*., 2016a). Lee *et al*., (2012b) review the broad range of TTC tactics used “to block tobacco-control policies and promote tobacco use” in LMICs. Gilmore *et al*. (2015) summarize how “industry systematically flaunts existing tobacco control legislation”, and uses “domestic litigation and international arbitration to bully LMICs”.

The agency power of the tobacco industry, to influence the FCTC process, is also well recognized (Morley *et al*., 2002; Wipfli, 2015). During negotiations, the industry secured membership on national delegations (Assunta and Chapman, 2006; Jin, 2014), sought to undermine WHO (WHO, 2000), and lobbied delegates (Otanez *et al*., 2009). Assunta (2012) discusses how the industry-funded International Tobacco Growers Association mobilized farmers to influence Conference of the Parties (COP4) negotiations, and defeated adoption of FCTC Articles 9 and 10 guidelines, and Articles 17 and 18 progress reports. Wipfli (2015) describes industry lobbying of delegates, presence on national delegations, hiring of private consultants and third party organizations to support industry positions, and diversion of attention to youth prevention and voluntary codes. She concludes that:

Despite its supposed exclusion from the FCTC process, the presence of the tobacco industry was felt throughout the negotiations. Their arguments formed the backbone of many of the most contentious debates among countries, and their impact is obvious in the final text of many key provisions in the treaty. (Wipfli, 2015: 52)

Overall, as Yach and Bettcher (2000) observed, the primary research focus remains on the “huge tobacco multinationals … attempting to manipulate globalisation trends in their favour”.

## An interdisciplinary research agenda on tobacco industry globalization

While tobacco industry globalization has received deserved attention within the public health community, resulting in a stronger evidence base for collective action, the above review points to opportunities for fuller understanding through an interdisciplinary research agenda. This can be achieved by drawing on two disciplines in which globalization has been a core concern: IPE and business studies.

### Clarifying definitions of tobacco industry globalization

As a first step, we believe a more precise definition of globalization is needed. The definitions of globalization used in the public health literature (focussing predominantly on policy influence, marketing, and market access and growth) imply an expanded capacity by the tobacco industry to assert influence, operate, and secure economic gains, over a wider geographical territory. However, it is difficult to delineate what is changing in the tobacco industry, whether this is new or distinct, and whether existing or new collective action is needed to govern its trajectory and impacts.

The work of Scholte (2008) is useful for distinguishing globalization from the terms *liberalization, universalization, westernization* and *internationalization*. What is novel about globalization, he argues, is “a shift in the nature of social space”:

The trans-territorial connections of globality are different from the inter-territorial connections of internationality. The transborder transactions of globality are different from the open-border transactions of liberality. The transplanetary simultaneity and instantaneity of supraterritoriality is different from the worldwideness of universality. The geographical focus of globality is different from the cultural focus of western modernity. Although globalisation … has some overlap with, and connections to, internationalisation, liberalisation, universalization and westernization, it is not equivalent to any of these older concepts and trends (Scholte, 2008: 1499).

This conceptualization is consistent with Dicken’s distinction between “internationalization” and “globalization”. *Internationalization* involves the “simple extension of economic activities across national boundaries. It is, essentially, a *quantitative* process which leads to a more extensive geographical pattern of economic activity” (Dicken, 2015). *Globalization* is *qualitatively* different, involving “not merely the geographical extension of economic activity across national boundaries but … the functional integration of such internationally dispersed activities” (Dicken, 2015). In this sense, internationalization and globalization co-exist, but “in ways which are highly uneven in space, in time and across economic sectors. Very few industries are truly and completely global although many display some globalizing tendencies” (Dicken, 2015). Much of what is currently claimed to be tobacco industry globalization falls under what Dicken (2015) describes as “nothing new”. For example, the practices of “state control over the market … gradually removed” (Gultekin-Karakas, 2015), use of “vehicles and themes to construct a tobacco culture in Asia” (Knight and Chapman, 2004b), development of “a lucrative market for mild, light, and ultra-low-tar cigarettes” (Assunta and Chapman, 2007) and “state-owned cigarette monopolies … taken over by the TTCs” (Connolly, 1992: 29) are describable with “pre-existent vocabulary” (Scholte, 2008: 1473).

What needs fuller understanding is the extent to which the tobacco industry is located within a “new geo-economy” (Dicken, 2015). To what extent have industry actors, or aspects of their operations, progressed beyond replication and expansion into new national markets, to the restructuring of their operations supraterritorially. Holden *et al*. (2010a) and Holden and Lee (2011) have begun to explore this trend in Latin America, including regional restructuring and TTC efforts to influence trade rules to support this operational logic. There is evidence that the global consolidation of tobacco leaf growing and processing, centred around three (Hammond, 1998) and then two dominant firms, is being accompanied by supraterritorial restructuring (Goger *et al*., 2014). Overall, appreciation of these definitional distinctions offers greater precision about the nature of the changes occurring in specific parts of the tobacco industry, and as a whole, and thus the extent to which true globalization is occurring.

### Measuring degree and variation in tobacco industry globalization

More precise indicators are needed to measure tobacco industry globalization. Rather than describing globalization as binary in occurrence, uniform across the industry, or linear in its trajectory, globalization can be studied as a process that is happening to varying degrees in different parts of the industry, at different geographical locations, with diverse features and varying impacts on production and consumption. We suggest measuring globalization by focusing on two types of indicators.

First, *firm-level indicators* measure changes in individual companies that suggest progression from national, to international, to global concerns. A key firm-level indicator is business type such as sole proprietorship, partnerships, corporations and cooperatives. Each type (and subtype) has different forms of ownership, governance, liability, regulatory burden and taxation. All have been found in the tobacco industry but, over time, corporations have dominated (Callard *et al*., 2005; Physicians for a Smoke-Free Canada, 2008). Changes to ownership, from state-owned to private enterprises via privatization and M&As, have been especially important in tobacco industry globalization. Firms that privatize, but do not “go large”, appear at risk of being swallowed up by other firms seeking regional or global economies of scale. Moreover, most TTCs are publicly-traded rather than private companies, with shareholders distributed worldwide. This form of ownership may facilitate globalization by enabling access to sufficiently large capital investment. There are notable exceptions such as JTI (partly state-owned) and the state-owned China National Tobacco Corporation. Analysis of the relationship between type of business and form of ownership, and global business strategy pursued, remains needed.

Type of organizational structure can also serve as firm-level indicators of globalization. A firm initially seeking to expand foreign markets will traditionally establish separate domestic and foreign divisions. As the firm’s business shifts increasingly to foreign markets, it may replicate its domestic operations in foreign markets (for example, multinational enterprise). As foreign markets grow even further, the firm may adopt a more decentralized organizational structure divided by function, production or service, customer or location (that is, transnational enterprise). There are many variations to the latter, to support a global business strategy, in terms of the distribution of assets, operations and human resources (Chee and Harris, 1998). A more globalized firm will, not only have substantial foreign operations, but hold a larger proportion of assets offshore, perhaps to reduce tax liability (Stulz, 1999).

Other indicators could be the diversity of senior management, levels of intra-firm trade, and distribution of a firm’s operations across different jurisdictions. A higher proportion of sales and earnings will not only be achieved through exports, but through overseas production by joint ventures, licensed manufacturers or factories abroad. This organizing principle can be understood in relation to a global production or supply chain (for example, manufacturing, R&D, raw materials) (van Hoeck *et al*., 2010). This type of data can be gleaned from company reports, as well as business and financial news sources. A particularly useful source is the United Nations Conference on Trade and Development (UNCTAD), which provides an “internationalization index” for the world’s top 100 non-financial TNCs, calculated as the number of (majority owned) foreign affiliates divided by number of all affiliates (UNCTAD, 2015). UNCTAD also compiles a “transnationality index” for the annual *World Investment Report* calculated as the average of three ratios: foreign assets to total assets; foreign sales to total sales; and foreign employment to total employment (UNCTAD, 2015). This measure has been comparatively applied to tobacco (Holden and Lee, 2009) and alcohol companies (Hawkins *et al*., 2016). *Fortune* magazine also provides revenue data for the world’s largest 500 companies (Global 500) list (Fortune, 2015). These data exclude smaller, but highly transnationalized, firms. Depending on the availability of standardized and longitudinal data, indicators such as the above could be used to develop a composite index of tobacco industry globalization.

A second way to measure tobacco industry globalization is *industry-level indicators*. These concern changes to the structure and activities of the industry as a whole. One potential indicator is the concentration ratio. Using the Herfindahl—Hirschman Index (HHI)^3^ as the most commonly accepted measure of market concentration, Hawkins *et al*. (2016) show that the tobacco industry in almost all countries has a very high concentration ratio, often the most concentrated sector in an economy. Further research is needed to calculate and analyze HHI scores for the tobacco industry by region and globally over time, to understand trends in concentration of the industry as a feature of globalization. This, in turn, can inform more detailed analysis of the factors behind these trends (for example, barriers to entry, business strategies), and the implications for global governance. The work of Philip Shepherd, on the concentration of cigarette manufacturing into an oligopoly in Latin America from the 1960s, is a useful starting point. He writes that stagnation of traditional markets prompted fierce competition for new markets, notably between US companies and BAT. By the late 1970s, a “two-tiered stratification of firms” (Shepherd, 1985) emerged, consisting of TTCs seeking regional expansion, and facilitated by barriers to entry (for example, economies of scale, brand awareness) and economic policies. Smaller firms (for example, Reetsma, Lorillard, Liggett) retreated to national markets and/or diversified into other products. There is a need to extend this analysis, beyond the 1980s, when tobacco industry globalization accelerated worldwide (Denniston, 2010).

Another set of useful industry-level indicators are offered through global value chain (GVC) analysis. This framework captures the interconnectedness of certain sectors in the world economy, including shifting patterns of tobacco trade and production. GVCs are “globally dispersed networks of firms and other institutional actors that coordinate to produce given goods or services for consumption” (Goger *et al*., 2014: 1). The framework is a promising conceptual tool to “understand how these networks are organized, reconfigured, and coordinated at different levels—national, regional, and global”, through “an examination of actors involved, stages of production in the chain, trade patterns, market dynamics, and governance structures to inform policy that promotes economic and social development”. Building on “more comprehensive understanding” of the tobacco industry in Malawi, Zimbabwe and Indonesia (Goger *et al*., 2014: 1), GVC analysis could be applied to other national, regional and global settings. For this purpose, a wide array of large-scale data gathering and analysis projects by key international organizations, such as the OECD-WTO Joint Trade in Value Added Database (TiVA), World Input-Output Database of the European Commission (Timmer, 2012), could be usefully drawn upon. Studies of the economic impact of GVCs, sponsored by the World Bank (Taglioni and Winkler, 2014) and International Monetary Fund (Saito *et al*., 2013), are also noteworthy. Such data help identify points in the tobacco GVC which are more or less globalized, and how this chain is distributed across different geographies. In addition, fuller understanding of the illicit tobacco trade, estimated to constitute around 11% of the world cigarette trade in 2008 (Joosens *et al*., 2009), could be similarly mapped using GVC analysis. Accurate data on volumes, sources and geographical flows are not possible, given the nefarious nature of the trade, and its passage through several jurisdictions before final sale. Nevertheless, using a GVC framework to integrate existing studies of selected national and regional jurisdictions will provide a clearer understanding of the increased interconnectedness of the illicit trade over time and space.

### Extending the temporal analysis of tobacco industry globalization

Analysis of tobacco industry globalization would benefit from greater attention to temporal dimensions of change, their location within particular historical timeframes, and the articulation of these with geographical factors. The literature does acknowledge differences in geographical focus over time. It remains unclear, however, how the pace of globalization has progressed or varied over time across different geographies. The work of Shepherd (1985) is again useful, in this respect, by documenting what might be considered as a first phase of tobacco industry globalization in Latin America in the 1960s. Building on this work, Lee *et al*. (2013) identify two subsequent phases of expansion, in Asia from the 1980s and Eastern Europe in the 1990s. Future research may examine a fourth phase, comprising the emergence of new TTCs, most likely from Asian companies adapting to foreign competition and seeking to globalize.

Importantly, not all firms will pursue a global strategy at the same time or pace, or even at all. Some may not be capable, or choose deliberately not, to globalize. First, there can be *non-globalization* whereby a firm focuses attention solely on the domestic market. We suggest classifying such firms, with a purely national orientation, as domestic tobacco companies. Second, where a firm is active in regional markets, in addition to the domestic market, there is a strategy of *semi-globalization*. For example, firms may initially focus on expanding activities regionally and, if successful and desirable, later expand operations (Rugman, 2005). Semi-globalization implies “neither extreme geographical fragmentation of the world in national markets nor complete integration” (Rugman and Verbeke, 2004: 6). Regional strategies should be viewed, in this sense, as a complement, rather than alternative, to domestic operations. Tobacco firms with this orientation can be classified as regional tobacco companies. Third, where a firm is active domestically, regionally, and beyond, these firms might be classified as TTCs engaging in *globalization*. Together, this suggests a more sophisticated conceptualization whereby domestic, regional and global business strategies can coexist and impact upon each other. Firms may pursue different strategies over time depending on diverse internal and external factors at play (Ghobadian *et al*., 2014).

An expanded historical timeframe of tobacco industry globalization would also benefit from several excellent histories of tobacco production and consumption, which offer important contextualization of contemporary trends (Kluger, 1996; Cox, 2000; Gately, 2001; Brandt, 2008; Proctor, 2011). Brandt (2008) observes that “[*n*]o one has followed globalization more closely or better understood its implications than the tobacco companies” (452). Analysis of tobacco industry globalization can build on these important works by, for example, relating existing patterns of leaf production to colonization (Benson, 2012), trends in the illicit trade to the history of marginalized populations, and emergence of TTCs to the spread of neo-liberalism from the late twentieth century.

### Diversifying units and levels of analyses

As described above, TTCs are the unit of analysis in most of the literature to date given the prominence of their activities and access to internal documents. Moreover, TTCs have largely been studied as unitary, separate and homogeneous actors operating at the national level. The business studies literature, sometimes written by industry insiders, is valuable for understanding the distinct way in which TTCs, given varied operating environments and organizational structures, have engaged with globalization. Ghoshal and Nohria (1993) evaluate 41 large companies (including RJ Reynolds, BAT and Swedish Match) to identify combinations of environment and structure that work better than others. Former BAT Head of Supply Chain Development, Andy Birtwistle, analysed the firm’s experiences reconfiguring its European and global supply chains (Godsell *et al*., 2010; van Hoeck *et al*., 2010).

Beyond TTCs, there are opportunities for new insights by expanding attention to other units and levels of analyses (see Table 1). Looking at other industry actors along the GVC—including national firms and state-owned enterprises, leaf growers and processers, and other sectors directly supporting TTC operations (for example, financiers, logistics, accountants, management consultants, advertising firms, legal representatives, wholesalers and retailers)—would provide fuller understanding of the diversity of globalization experiences. A further set of actors support the political and economic interests of the industry worldwide including industry associations, chambers of commerce, third parties, manufacturers of tobacco-related products (for example, matches, lighters, cigarette paper), media and industry funded groups. The comprehensive mapping of industry actors in some national settings (Granero *et al*., 2004; Li, 2012) could be extended to the global level. There is much to be learned from comparative analysis of the motivations and strategies for globalization by different industry actors. By comparing firm-level indicators discussed above, we may answer such questions as why some pursue globalization while others do not; what different business strategies they use and why; and whether and why some are more successful than others.

For identifying why an actor may pursue globalization in the first place, the work of Dunning and colleagues is useful. TTCs may be understood as seekers of natural resources, new markets, efficiency or strategic assets. Firms may choose to adopt a global business strategy for one or more of these reasons (Yip, 1998; Dunning and Lundan, 2008a, b). There are also key advantages of a global over domestic business strategy. This may explain, for example, different patterns of vertical integration in leaf production (for an analysis of the Brazilian case see, for example, Ladu, 2014; Tuinstra, 2014).

Other business literature helps us to better understand and compare specific strategies by firms pursuing globalization. The most direct strategy is exporting to customers outside of a firm’s domestic market. Alternatively, the firm may engage in indirect exports, for example, by licensing of another firm to supply a foreign market. If a firm wishes to be physically closer to foreign customers, then they may engage in foreign direct investment (FDI) including strategic alliances with other firms (for example, joint venture), M&As and setting up a new venture (that is, greenfield investments). Internally, firms may reorient towards foreign markets, gain further efficiencies and enhance access to inputs in several ways. Firms may also recruit human capital (for example, business executives, consultants and management) to gain specialist knowledge and acquire new technology and knowledge (for example, leaf production, manufacturing machinery) to improve quality and productivity. Or firms may adapt or develop new products (for example, low tar cigarettes) that appeal to target consumers. Firms may engage in direct and indirect marketing to increase brand recognition. Finally, firms may establish new distribution channels including legal and illegal means. Some of these strategies have been documented, in relation to the tobacco industry, but only in selected contexts and timeframes. For example, Shepherd (1985) examines the replacement of national tobacco companies in Latin America by TTC subsidiaries by the 1980s, achieved through a combination of legal and illegal strategies that resulted in control of the entire product cycle. To date, there has been no systematic, and limited comparative, analysis across different industry actors, contexts and timeframes to explain which strategies have been pursued where, by whom and why.

The above can also contribute to deeper analysis of the industry, as a whole, as the unit of analysis. As described above, there are a broad range of industry-level indicators to measure its changing nature and dynamics amid globalization. This would especially benefit growing interest in comparative analysis of tobacco with other health-related industries including alcohol, food and drink, and pharmaceuticals (Wiist, 2010; Hawkins *et al*., 2016; Kenworthy *et al*., 2016). How does tobacco industry globalization compare with other industries in form, dynamics, trajectory and, from a public health perspective, regulatory needs? Such comparisons would also help us to analyse the extent to which the tobacco industry should be treated differently to other health-harming industries.

### Understanding the interplay between structural and agency power

As indicated in the literature review above, the vast majority (96%) of the reviewed papers focus on agency power—that is, the ability of firms to act independently in ways that achieve desired ends—by tobacco firms. The agency power of TTCs has undoubtedly been a key driver of tobacco industry globalization, and much remains to be understood about how the industry has asserted agency power in diverse venues, through direct and indirect actions, and over time. For example, TTCs have increasingly exploited “judicialized” forms of global governance. Eckhardt *et al*. (2015) analyse TTCs lobbying within the context of the WTO and show how this has increased opposition to stronger tobacco control measures on the side of developing countries. Similarly, there has been growing study of how the industry has strategically framed tobacco control issues in ways favourable to its interests. This capacity has been well-documented in relation, for example, to the scientific evidence on tobacco and health science (Bero, 2003; Hurt, *et al*, 2009); the “accommodation” of smokers in public smoking restrictions (Dearlove *et al*., 2002; Sebrie and Glantz, 2007); and the need for “constructive solutions”, “sensible regulation” and “good governance” (Smith *et al*., 2009). More research is needed to extend framing theory (Schön and Rein, 1996) to industry efforts to shape the governance of globalization. How TTCs frame regimes on, for example, trade and investment (Fooks and Gilmore, 2013), anti-trust, financial reporting, taxation and criminal activity warrant fuller attention.

It is this need to understand both industry adaptation to, and its efforts to shape, globalization that points to the interplay between agency and structural power. Holden and Lee (2009) suggest that agency power can be exerted to promote certain ideas that frame issues and policy responses, and thus shape the consequent “rules of the game”. Once embedded in global governance, the ideas themselves come to constitute structural power, delimiting what is seen as acceptable or unacceptable, possible or impossible. The choices of other actors are structured and there is less need for agency power. In the tobacco industry, globalization has brought dominance by an oligopoly, with enormous resources to exert agency power by selecting, exploiting and, indeed, shaping the institutions of global governance. With extensive resources and worldwide reach, TTCs have opportunities to exploit the complexity and fragmentation of global governance mechanisms, and shape them to serve their interests. Baker *et al*. (2014: 66), for instance, show how the liberalization of trade and investment allows TTCs, and other “transnational risk commodity corporations”, to “rapidly move investments, technologies, production capacity, raw materials and final products across borders and thereby drive risk commodity consumption transnationally”.

At the same time, TTC power may not be as impermeable as often described. Forty percent of world production is still accounted for by state-owned enterprises (Hogg *et al*., 2015). The rise of Asian tobacco companies, and continued restructuring of the industry and individual firms, supports Farnsworth’s (2004) assertion that agency and structural power varies over time. The greater opportunity by industry actors to “venue shop”, amid increased regime complexity, illustrates the above. Actors may shop for the policy-making forum or legal institution where they are most likely to obtain a favourable outcome, and attempt to *shift* decision-making to that forum. Evidence suggests that venue shopping firms are generally TNCs “sourcing from abroad or firms with foreign subsidiaries, that are confronted with an unresponsive home government and use the opportunities of the multiple [global] venues available” (Eckhardt and De Bièvre, 2015: 513). This logic appears to apply to the tobacco industry, but more research is needed on how TTCs have promoted the formation of alternative venues, including the reframing of issues (Baumgartner and Jones, 1991) such as packaging (intellectual property rights), tariffs (market access), illicit trade (law and order) and farming (rural employment), in ways that allow the shifting of public health issues to these venues. Jarman (2014), for example, argues that shifting public health debates to “exclusionary” trade and investment venues shapes political conflict by influencing “which voices are heard or excluded from a particular debate”.

As well as benefitting from regime complexity, TTCs may seek to shape emerging forms of global governance through political strategies and influence during trade and investment negotiations. Research shows that the largest and most productive firms benefit from such agreements via increased trade, investment and production opportunities (Melitz, 2003). These firms also have greater means to influence policy outcomes and restructure their production processes accordingly. TTCs are among the world’s largest and most productive firms, and are well-placed to influence negotiations and reap their benefits. Building on the study of firm involvement in trade agreement formation (Chase, 2003; Dür, 2007; Eckhardt and Poletti, 2015), and tobacco industry influence of negotiations within the Andean Pact and Central American Common Market (Holden *et al*., 2010a; Holden and Lee, 2011) and WTO accession (Holden *et al*., 2010b), there is need for analysis of other trade and investment negotiations. This includes indirect influence through front and proxy groups such as the International Chamber of Commerce (Hakim, 2015).

### Implications for global health governance

Widespread recognition of tobacco industry globalization, and its population health impacts, prompted collective action efforts including adoption of the FCTC. The FCTC negotiation process, and subsequent implementation, was remarkably successful in shifting public debate, from a focus on personal responsibility and behavioural change, to locating tobacco control within the domain of global governance. However, because the treaty was ostensibly prompted by public health concerns about the expansion of the tobacco industry into emerging markets, and the consequent rise in tobacco-related morbidity and mortality, policy measures have largely focused on transferring or scaling up tobacco control measures used in traditional markets. There is far less understanding of the distinct governance challenges posed by globalization. As Bollyky and Fidler write,

the FCTC makes few demands on parties to address international aspects of tobacco control. It contains no obligations concerning licit international trade and investment, cross-border advertising, assistance for developing countries, or strong dispute settlement. The FCTC requires parties to monitor and prevent illicit trade in tobacco products within their territories, with a promise to consider a protocol [agreed but not yet in effect] on international cooperation on cigarette smuggling at a later date …. the FCTC’s binding provisions focus on domestic tobacco-control measures that were effective, but did not require a treaty for countries to implement. (Bollyky and Fidler, 2015)

This article demonstrates how public health research can be supplemented by an interdisciplinary agenda that provides clearer definition and measurement, more diverse units and levels of analyses, location within an historical timeframe, and understanding of the interaction between agency and structural power. There is need for fuller understanding of how globalization has elicited adaptation by different industry actors, across different institutional settings and geographies, over time. Expanded research would offer important insights for strengthening global governance to address the increasingly transnational nature of the industry. This would include stronger implementation of FCTC Article 5.3, supply-side measures, and policy coherence with other global governance spheres.

The latter point reminds us that the realms of global governance relevant to tobacco control go far beyond the public health sphere (Collin, 2012). Indeed, there are forms of global *economic* governance that effectively serve tobacco industry interests. This is because compliance with many of the rules of international economic organizations, such as the World Bank and WTO, are backed by compulsion (for example, lending conditions) or “hard” law (for example, trade sanctions). In contrast, international social organizations mainly rely on “soft” law (for example, codes of conduct). The FCTC is a binding international treaty but lacks mandatory enforcement mechanisms or punitive measures for noncompliance. On the one hand, therefore, globalization prompts collective action responses by state and non-state actors that together constitute expanding forms of global governance. On the other hand, global governance is evolving in a messy and uneven manner, including overlapping spheres of operation, regulatory gaps, unclear lines of authority and even competing goals. Tobacco industry globalization has occurred amid this evolving context.

Moreover, conflicts between norms in international law, such as obligations under the FCTC and WTO agreements, remain unresolved (McGrady, 2012; Eckhardt *et al*., 2015). Following WTO’s establishment in 1995, attention to the implications for tobacco control has focused on industry use of such agreements to press for increased market access (Shaffer *et al*., 2005) or prevent stronger regulation. For example, the Agreement on Trade-Related Intellectual Property Rights, General Agreement on Trade in Services and Agreement on Technical Barriers to Trade have all been used to challenge the adoption of plain packaging, ingredients disclosure and labelling (Gervais, 2010; Frankel and Gervais, 2013; Marsoof, 2013; Eckhardt *et al*., 2015). Investor–state dispute settlement gives TTCs legal standing to directly challenge tobacco control measures under investment treaties. This proliferation of bilateral and regional agreements, and stalling of WTO negotiations, has made the trade and investment regime even more complex (Alter and Meunier, 2009). Despite the attempt to construct an overarching multilateral institution in the form of the WTO, the numerous and overlapping bilateral, regional, plurilateral and multilateral treaties form a “spaghetti bowl” rather than ordered hierarchy of institutions and agreements (Bhagwati, 1995). A fuller understanding of tobacco industry globalization, and the related ability to exploit such complexity via agency and structural power, will be crucial to future tobacco control efforts.

## Supplementary Material

Supplementary Material

Supplementary Table 1

## Figures and Tables

**Table 1:** Units and levels of analysis to study tobacco industry globalization

*Unit of analysis*	*Examples*
individual	adult smokers, senior executives, tobacco farmers, policymakers, lobbyists, ethnic minorities, smugglers, industry consultants
group	diaspora, board of directors, transit agents, corporate relations departments
organization	firms, industry associations, think tanks, WTO, International Chamber of Commerce
artifact	business strategy documents, advertisements, social media content, packaging and labelling, nicotine delivery products
geographical area	countries, free trade zones, low-income countries, regional markets
social interaction	lobbying, CSR initiatives, donations, legal actions, public hearings, public relations, meetings and conferences
*Level of analysis*	
organizational	firms, front groups, government ministries, tobacco industry
state	countries
system	global tobacco market (oligopoly), global supply chain, world economy, criminal network

## Footnotes

1 Before the large-scale release of internal industry documents in the late 1990s, most of the reviewed literature broadly examine industry activities in LMICs as the unit of analysis (Stebbins, 1987; Connolly, 1992; MacKay and Crofton, 1996).

2 Some of the papers reviewed use more than one level of analysis.

3 The HHI is calculated by squaring, and then adding together, the market share of all the firms competing in a particular sector.
