# Supplementary material for: Tobacco industry globalization and global health governance: towards an interdisciplinary research agenda
Source: Palgrave Commun. Author manuscript; Available in PMC 2017 Apr 28. (PMC5409523; doi:10.1057/palcomms.2016.37)
Supplement: Supplementary Material [file NIHMS818558-supplement-Supplementary_Material.doc]

**Table 1 References**

1. Currie K, Ray L (1984) Going up in smoke: the case of British American tobacco in Kenya. *Social Science & Medicine* 19(11): 1131-1139.
2. Stebbins KR (1987) Tobacco or health in the Third World: a political economy perspective with emphasis on Mexico. *International Journal of Health Services* 17 (3): 521-536.
3. Nichter M, Cartwright E (1991). Saving the children for the tobacco industry. *Medical Anthropology Quarterly* 5 (3):236-56.
4. Connolly GN (1992) Worldwide expansion of transnational tobacco industry. *JNCI Monographs* 12: 29-35.
5. Mackay J (1994) The tobacco problem: commercial profit versus health – the conflict of interest in developing countries. *Preventive Medicine* 23(4): 535-8.
6. Stebbins KR (1994) Making a killing south of the border: transnational cigarette companies in Mexico and Guatemala. *Social Science and Medicine* 38(1): 105-15.
7. Mackay J, Crofton J (1996). Tobacco and the developing world. *British Medical Bulletin* 52(1): 206-21.
8. Hammond R. (1998) Consolidation in the tobacco industry. *Tobacco Control* 7: 426-8.
9. Chantornvong S, Collin J, Dodgson R, Lee K, McCargo D, Seddon D, Vaughan P, Woelk G (2000). Political economy of tobacco control in low-income and middle-income countries: lessons from Thailand and Zimbabwe. *Bulletin of the World Health Organization* 78(7): 913-9.
10. Honjo K and Kawachi I (2000). Effects of market liberalisation on smoking in Japan. *Tobacco Control* 9(2): 193-200.
11. O’Sullivan B and Chapman S (2000). Eyes on the prize: transnational tobacco companies in China 1976-1997. *Tobacco Control* 9(3): 292-302.
12. Stebbins KR (2001). Going like gangbusters: transnational tobacco companies “making a killing” in South America. *Medical Anthropology Quarterly* 15(2): 147-70.
13. Collin J, Lee K and Bissell K (2002) The Framework Convention on Tobacco Control: The politics of global health governance. *Third World Quarterly* 23(2): 265-82.
14. Hilamo H (2003). Tobacco industry strategy to undermine tobacco control in Finland. *Tobacco Control* 12(4): 414-23.
15. Szilagyi T, Chapman S (2003a). Hungry for Hungary: examples of tobacco industry’s expansionism. *Central European Journal of Public* *Health* 11(1): 38-43.
16. Szilagyi T, Chapman S (2003b). Tobacco industry efforts to keep cigarettes affordable: a case study from Hungary. *Central European Journal of Public Health* 11(4): 223-8.
17. Gilmore AB, MacKee M (2004a). Moving East: how the transnational tobacco industry gained entry to the emerging markets of the former Soviet Union – part I: establishing cigarette imports. *Tobacco Control* 13(2): 143-50.
18. Gilmore AB, MacKee M (2004b). Moving East: how the transnational tobacco industry gained entry to the emerging markets of the former Soviet Union – part II: an overview of priorities and tactics used to establish a manufacturing presence. *Tobacco Control* 13(2): 151-60.
19. Gilmore AB, MacKee M (2004c). Tobacco and transition: an overview of industry investments, impact and influence in the former Soviet Union. *Tobacco Control* 13(2): 136-42.
20. Knight, J. and S. Chapman (2004a). Asia is now the priority target for the world anti-tobacco movement’: attempts by the tobacco industry to undermine the Asian anti-smoking movement. *Tobacco Control* 13(Suppl. 2): ii30-ii36.
21. Knight J, Chapman S (2004b). ‘Asian yuppies…are always looking for something new and different’: creating a tobacco culture among young Asians. *Tobacco Control* 13(Suppl2): ii22-9.
22. Lambert A, Sargent JD, Glantz SA, Ling PM (2004). How Philip Morris unlocked the Japanese cigarette market: lessons for tobacco control. *Tobacco Control* 13(4): 379-87.
23. Lawrence S, Collin J (2004). Competing with kreteks: transnational tobacco companies, globalization, and Indonesia. *Tobacco Control* 13(suppl 2): ii96-ii103.
24. Lee K, Gilmore AB, Collin J (2004). Breaking and re-entering: British American Tobacco in China 1979-2000. *Tobacco Control* 13 (suppl. 2): ii88-95.
25. Mackenzie R, Collin J, Sriwongcharoen K, Muggli ME (2004). “If we can just stall new unfriendly legislations, the scoreboard is already in our favour”: transnational tobacco companies and ingredients disclosure in Thailand. *Tobacco Control* 13 (Suppl 2): ii79-87.
26. Szilagyi T, Chapman S (2004). Tobacco industry efforts to erode tobacco advertising controls in Hungary. *Central European Journal of Public Health* 12(4): 190-6.
27. Tong EK, Glantz SA (2004). ARTIST (Asian regional tobacco industry scientist team): Philip Morris’ attempt to exert a scientific and regulatory agenda on Asia. *Tobacco Control* 13 (Suppl 2): ii18-24.
28. Gilmore AB, Radu-Loghin C, Zatushevki I, MacKee M (2005). Pushing up smoking incidence: plans for a privatised tobacco industry in Moldova. *Lancet* 365(9467): 1354-9.
29. Sebrie EM, Barnoya J, Perez-Stable EJ, Glantz SA (2005). Tobacco industry successfully prevented tobacco control legislation in Argentina. *Tobacco Control* 14(5): e2.
30. Assunta M, Chapman S (2007). The lightest market in the world: light and mild cigarettes in Japan. *Nicotine and Tobacco Research* 10(5): 803-10.
31. Gilmore A, Collin J, Townsend J (2007). Transnational tobacco company influence on tax policy during privatization of a state monopoly: British American Tobacco and Uzbekistan. *American Journal of Public Health* 97(11): 2001-9.
32. Zhong F, Yano E (2007). British American Tobacco’s tactics during China’s accession to the World Trade Organization. *Tobacco Control* 16(20): 133-7.
33. LeGresley E, Lee K, Muggli ME, Patel P, Collin J, Hurt RD (2008). British American Tobacco and the “insidious impact of illicit trade” in cigarette smuggling across Africa. *Tobacco Control* 18(5): 339-46.
34. MacKenzie R, Collin J (2008). “A good personal scientific relationship”: Philip Morris scientists and the Chulabhorn Research Institute, Bangkok. *PLoS Medicine* 5(12): 1737-48. doi:10.1371/journal.pmed.0050238.
35. McDaniel PA, Intinarelli G, Malone RE (2008). Tobacco industry issues management organizations: creating a global corporate network to undermine public health. *Global Health* 4: 2. doi:10.1186/1744-8603-4-2.
36. Muggli ME, Lee K, Gan Q, Ebbert JO, Hurt RD (2008). “Efforts to reprioritize the agenda” in China: British American Tobacco’s efforts to influence public policy on secondhand smoke in China. *PLoS Medicine* 5(12): 1729-69.
37. Nakkash R, Lee K (2008). Smuggling as the “key to a combined market”: British American Tobacco in Lebanon. *Tobacco Control* 17: 324-31.
38. Lee K, Carpenter C, Challa C, Lee S, Connolly GN, Koh HK (2009). The strategic targeting of females by transnational tobacco companies in South Korea following trade liberalization. *Global Health* 5: 2. doi: 10.1186/1744-8603-5-2.
39. Holden C, Lee K (2009). Corporate power and social policy: the political economy of the transnational tobacco companies. *Global Social Policy* 9(3): 328-54.
40. Nakkash R, Lee K (2009). The tobacco industry’s thwarting of marketing restrictions and health warnings in Lebanon. *Tobacco Control* 18(4): 310-6.
41. Otanez, MG, Mamudu, HM, Glantz SA (2009). Tobacco companies’ use of developing countries’ economic reliance on tobacco to lobby against global tobacco control: the case of Malawi. *American Journal of Public Health* 99(10): 1759-71.
42. Sebrie EM, Schoj V, Glantz SA (2009). Smokefree environments in Latin America: on the road to real change? *Prevention and Control* 3(1): 21-35.
43. Freeman B, Chapman S (2010). British American Tobacco on Facebook: undermining Article 13 of the global World Health organization Framework convention on Tobacco Control. *Tobacco Control* 19(3): e1-9.
44. Krasovsky KS. (2010). “The lobbying strategy is to keep excise as low as possible” – tobacco industry excise taxation policy in Ukraine. *Tobacco Industry Discourse* 8: 10.
45. Stanton CR, Chu A, Collin J, Glantz SA (2010). Promoting tobacco through the international language of dance music: British American Tobacco and the Ministry of Sound. *European Journal of Public Health* 2(1): 21-28.
46. Chu A, Jiang N, Glantz SA (2011). Transnational tobacco industry promotion of the cigarette gifting custom in China. *Tobacco Control* 20(4): e3.
47. Holden C, Lee K (2011). A major lobbying effort to change and unify the excise structure in six Central American countries”: how British American Tobacco influenced tax and tariff rates in the Central American Common Market. *Globalization and Health* 7: 15. doi:10.1186/1744-8603-7-15.
48. Bialous SA, Peeters S (2012). A brief overview of the tobacco industry in the last 20 years. *Tobacco* *Control* 21: 92-94.
49. Charoenca N, Mock J, Kungskulniti N, Preechawong S, Kojetin N, Hamann SL (2012). Success counteracting tobacco company interference in Thailand: an example of FCTC implementation for low- and middle-income countries. *International Journal of Environmental Research and Public Health* 9(4): 1111-34. doi: 10.3390/ijerph9041111.
50. Gilmore AB (2012). Understanding the vector in order to plan effective tobacco control policies: an analysis of contemporary tobacco industry materials. *Tobacco Control* 21(2): 119-26.
51. Hurt RD, Ebbert JO, Achadi A, Croghan IT (2012). Roadmap to a tobacco epidemic: transnational tobacco companies invade Indonesia. *Tobacco Control* 21(3): 306-12.
52. Lee SY, Ling PM, Glants SA (2012). The vector of the tobacco epidemic: tobacco industry practices in low and middle-income countries. *Cancer Causes and Control* 23(01): 117-29.
53. Shrinae R, Smith K, Ross H, Silver KE, Williams S, Gilmore A (2012). Tobacco industry manipulation of tobacco excise and tobacco advertising policies in the Czech Republic: an analysis of tobacco industry documents. *PLoS Medicine* 9(6): e1001248.
54. Weishaar H, Collin J, Smith K, Gruning T, Mandal S, Gilmore A (2012). Global health governance and the commercial sector: a documentary analysis of tobacco company strategies to influence the WHO framework convention on tobacco control. *PLoS Medicine* 9(6): 31001249.
55. Bump JB, Reich MR (2013). Political economy analysis for tobacco control in low- and middle-income countries. *Health Policy & Planning* 28(2): 123-33. doi:10.1093/heapol/czs049.
56. Fooks G, Gilmore A (2013) International trade law, plain packaging and tobacco industry political activity: the Trans-Pacific Partnership. Tobacco Control. doi:10.1136/tobaccocontrol-2012-050869
57. Lee S, Holden C, Lee K (2013). Are transnational tobacco companies’ market access strategies linked to economic development models? A case study of South Korea. *Global Public Health* 8(4): 435-48.
58. Lunze K, Migliorini L (2013). Tobacco control in the Russian Federation – a policy analysis. *BMC Public Health* 13:64.
59. Bake P, Kay A, Walls H (2014). Trade and investment liberalization and Asia’s noncommunicable disease epidemic: a synthesis of data and existing literature. *Global Health* 10: 66.
60. Crosbie E, Gonzalez M, Glantz SA (2014). Health preemption behind closed doors: trade agreements and fast-track authority. *American Journal of Public Health* 104(9): e7-e13.
61. Lee S, Lee K, Holden C (2014). Creating demand for foreign brands in a “home run market”: tobacco company tactics in South Korea following market liberalization. *Tobacco Control* 23(3): e8.
62. Savell E, Gilmore AB, Fooks G (2014). How does the tobacco industry attempt to influence marketing regulations? A systematic review. *PLoS One* 9(2): e87389.
63. Skafida V, Silver KE, Rechel BP, Gilmore AB (2014). Change in tobacco excise policy in Bulgaria; the role of tobacco industry lobbying and smuggling. *Tobacco Control* 23(e1): e75-84. doi:10.1136/tobaccocontrol-2012-050600.
64. Drope, J. Chavez JJ (2015). Complexities at the intersection of tobacco control and trade liberalization: evidence from Southeast Asia. *Tobacco Control* 24(e2): e128-36.
65. Eckhardt J, Holden C, Callard CD (2015). Tobacco control and the World Trade Organization: mapping member states’ positions after the framework convention on tobacco control. *Tobacco Control.* doi:10.1136/tobaccocontrol-2015-052486.
66. Gilmore AB, Fooks G, Drope J, Bioalous SA, Jackson RR (2015). Exposing and addressing tobacco industry conduct in low-income and middle-income countries. *Lancet* 385(9972): 1029-43.
67. Gultekin-Karakas D (2015). Can demand-side policies stop the tobacco industry’s damage? Lessons from Turkey. *Global Public Health* 20(7): 777-93.
68. Petticrew M, Lee K, Ali H, Nakkash R (2015). “Fighting a hurricane”: tobacco industry’s efforts to counter the perceived threat of Islam. *American Journal of Public Health* 105(6): 1086-93.
69. MacKenzie R, Lee K, LeGresley E. (2015). To “enable our legal product to compete effectively with the transit market’: British American Tobacco’s strategies in Thailand following the 1990 GATT dispute. *Global Public Health* 21:1-15.
70. Delobelle P, Sander D, Puoane T, Freudenberg N (2016). Reducing the role of the food, tobacco, and alcohol industries in noncommunicable disease risks in South Africa. *Health education and Behaviour* 43(Suppl1): 70S-81S.
71. Peeters S, Costa H, Stuckler D, MacKee M, Gilmore AB (2016). The revision of the 2014 European tobacco products directive: an analysis of the tobacco industry’s attempts to “break the health silo”. *Tobacco Control* 25(1): 108-17.
72. Smith J, Thompson S, Lee K (2016). ”Public enemy no. 1”: Tobacco industry funding for the AIDS response. *SAHARA J* 13(1): 41-52.
73. Smith J, Thompson S, Lee K (2016). The Atlas network: a “strategic ally” of the tobacco industry. *International Journal of Health Planning and Management*. doi:10.1002/hpm.2351
